# Epidemiology of Cardiopulmonary Arrest and Outcome of Resuscitation in PICU Across China: A Prospective Multicenter Cohort Study

**DOI:** 10.3389/fped.2022.811819

**Published:** 2022-04-28

**Authors:** Xin Ding, Gang Liu, Suyun Qian, Jiansheng Zeng, Ying Wang, Jianping Chu, Qing Chen, Jianli Chen, Yuanyuan Duan, Danqun Jin, Jiaotian Huang, Xiulan Lu, Yanmei Guo, Xiaona Shi, Ximin Huo, Jun Su, Yibing Cheng, Yi Yin, Xiaowei Xin, Zhengyun Sun, Shaodong Zhao, Hongjun Miao, Zixuan Lou, Jun Li, Jinghui Jiang, Shengying Dong

**Affiliations:** ^1^Department of Pediatric Intensive Care Unit, National Center for Children's Health, Beijing Children's Hospital, Capital Medical University, Beijing, China; ^2^Department of Pediatric Intensive Care Unit, Xi'an Children's Hospital, Xi'an, China; ^3^Department of Pediatric Intensive Care Unit, Guiyang Maternal and Child Health Care Hospital, Guiyang, China; ^4^Department of Pediatric Intensive Care Unit, Anhui Children's Hospital, Hefei, China; ^5^Department of Pediatric Intensive Care Unit, Children's Hospital of Hunan Province, Changsha, China; ^6^Department of Pediatric Intensive Care Unit, Hebei Children's Hospital, Shijiazhuang, China; ^7^Department of Pediatric Intensive Care Unit, Zhengzhou Children's Hospital, Zhengzhou, China; ^8^Department of Pediatric Intensive Care Unit, Shandong Provincial Hospital Affiliated to Shandong First Medical University, Jinan, China; ^9^Department of Pediatric Intensive Care Unit, Children's Hospital of Nanjing Medical University, Nanjing, China; ^10^Jinan Children's Hospital, Jinan, China; ^11^Department of Pediatric Intensive Care Unit, Liaocheng People's Hospital, Liaocheng, China

**Keywords:** cardiopulmonary arrest, cardiopulmonary resuscitation, critical ill children, multicenter study, China

## Abstract

**Objective:**

To investigate the epidemiology and the effectiveness of resuscitation from cardiopulmonary arrest (CPA) among critically ill children and adolescents during pediatric intensive care unit (PICU) stay across China.

**Methods:**

A prospective multicenter study was conducted in 11 PICUs in tertiary hospitals. Consecutively hospitalized critically ill children, from 29-day old to 18-year old, who had suffered from CPA and required cardiopulmonary resuscitation (CPR) in the PICU were enrolled (December 2017–October 2018). Data were collected and analyzed using the “in-hospital Utstein style.” Neurological outcome was assessed with the Pediatric Cerebral Performance Category (PCPC) scale among children who had survived. Factors associated with the return of spontaneous circulation (ROSC) and survival at discharge were evaluated using multivariate logistic regression.

**Results:**

Among 11,599 admissions to PICU, 372 children (3.2%) had CPA during their stay; 281 (75.5%) received CPR, and 91 (24.5%) did not (due to an order of “Do Not Resuscitate” requested by their guardians). Cardiopulmonary disease was the most common reason for CPA (28.1% respiratory and 19.6% circulatory). The most frequent initial dysrhythmia was bradycardia (79%). In total, 170 (60.3%) of the total children had an ROSC, 91 had (37.4%) survived till hospital discharge, 28 (11.5%) had survived 6 months, and 19 (7.8%) had survived for 1 year after discharge. Among the 91 children who were viable at discharge, 47.2% (43/91) received a good PCPC score (1–3). The regression analysis results revealed that the duration of CPR and the dose of epinephrine were significantly associated with ROSC, while the duration of CPR, number of CPR attempts, ventricular tachycardia/ventricular fibrillation (VT/VF), and the dose of epinephrine were significantly associated with survival at discharge.

**Conclusion:**

The prevalence of CPA in critically ill children and adolescents is relatively high in China. The duration of CPR and the dose of epinephrine are associated with ROSC. The long-term prognosis of children who had survived after CPR needs further improvement.

## Introduction

Cardiopulmonary arrest (CPA) is a sudden life-threatening event that could cause mortality, and cardiopulmonary resuscitation (CPR) is its only first aid. CPA is a critical condition in children and primarily occurs in the pediatric intensive care unit (PICU). The in-hospital mortality of children under CPA in the PICU after receiving CPR is between 38 and 69.6% (1, 2), which is considerably higher than the mortality of PICU admissions (2–3%) in a 4-year follow-up study (3). A standard CPR has been introduced across the world, and this standard has improved the effectiveness and prognosis post-CPR. However, the major indexes, such as returning of spontaneous circulation (ROSC) and survival rate, vary among different studies.

The Utstein style is a uniformly defined pattern of data collection and reporting. This type of reporting has been regarded as a convenient tool for comparing results of resuscitation among different studies but has been less frequently used in pediatric research. The first observational multicenter survey of pediatric in-hospital CPA in mainland China by Utstein style was conducted in 2013. The results revealed that, among the PICU admissions, the incidence of CPA was 5.5%, and the rates of ROSC and survival to hospital discharge were 60.3 and 25.5%, respectively (4). In the past two decades, training programs on basic life support (BLS) and pediatric advanced life support (PALS) have been conducted across China. The government investment in medical resources has increased, but the knowledge of CPA and CPR in China urgently needs to be updated. Therefore, we conducted this multicenter study using the Utstein style to analyze the epidemiologic characteristics of CPA and to determine the factors associated with improved outcomes after CPR in the PICU.

## Methods

### Multicenter Settings

The Beijing Children's Hospital was the organizing center and was responsible for this study. All 11 participating PICUs were in tertiary children's hospitals or general hospitals in north, central, east, southwest, and northwest China. These hospitals have well-established code teams and life support equipment.

All medical staff involved in this study were well trained in bedside CPR and were familiar with BLS, PALS, and Utstein style. A staff was designated in each center to check and collect data by using the Utstein chart. All accurate and qualified data were sent to the Beijing Children's Hospital to establish the central database and for analysis.

### Study Design

This study is a prospective observational multicenter study. Upon PICU admission, the guardians of critically ill children were required to sign a written medical directive in case of CPA to enable the medical staff to initiate CPR immediately. When CPA occurred, children whose guardians requested “Do Not Resuscitate (DNR)” most likely only received epinephrine or electric defibrillation with or without tracheal intubation for ventilation but without chest compression.

The inclusion criteria were children aged between 29-day old and ≤18-year old who were admitted to the PICU from 1 December 2017 to 31 October 2018 and who underwent CPA and CPR. Children whose CPA occurred outside the PICU, who were assigned DNR on the first CPA after PICU admission (including children with insufficient medical therapy when CPA occurred), or who were hospitalized in cardiac intensive care units for perioperative monitoring were excluded.

The standard process of CPR was referred to in the American Heart Association (AHA) guidelines of BLS and PALS (5), such as chest compression, open airway and ventilation support, and other interventions, including endotracheal intubation, vasoactive drugs, and defibrillation. The number of CPR attempts referred to how many rounds of CPR were performed. One round of CPR was a period from initiation to the end, which includes ROSC or death.

When CPA occurred repeatedly, only the first CPA after PICU admission was recorded and analyzed. All children were included as long as they received CPR. No specific duration for CPR was required.

Cardiopulmonary resuscitation is the only medical treatment for CPA, and no absolute contraindication of such a process has been identified. However, if thoracic deformities, open thoracic injuries, pericardial tamponade, and blood pneumothorax exist, parents should be fully informed in advance of the pros and cons of CPR under such circumstances. The final decision of whether to implement CPR was made by guardians.

This study had been approved by the Ethics Committee of the Beijing Children's Hospital.

### Data Collection

Data collected included age, gender, body weight, measures before resuscitation (e.g., endotracheal intubation, cardiac pacemaker, and electrocardiogram [ECG] monitoring), primary diagnosis, initial heart rhythm, time of CPR start-up, duration of CPR, number of CPR attempts, the dose of epinephrine, and use of sodium bicarbonate (e.g., the total number of doses and delivery method).

The prognosis of the neurological system of the children was evaluated using the Pediatric Cerebral Performance Category (PCPC) score, which has six categories: normal in neurodevelopmental and intelligence of children of the same age; mild disability; moderate disability; severe disability; coma/vegetative state; and death.

### Follow-Up Visit and Major Index

The children's guardians were informed in detail that the children would be followed up after discharge. The person-in-charge of the Beijing Children's Hospital team collected the phone number of every surviving child's guardian every month and followed them up by phone on the first 6 months and 1 year after discharge to inquire about the child's condition.

The main indexes used to assess the effect of CPR included ROSC (i.e., heartbeat recovery, palpable pulse, and spontaneous recovery ≥20 min), survival rate (24 h after CPR, on hospital discharge day, and first 6 months and 1 year after hospital discharge), and PCPC score.

### Statistical Methods

SPSS version 22.0 was used for the statistical analysis. Pearson's chi-square test was used to analyze differences between categorical data groups. If the value was <5 or the sum was <20, Fisher's exact test analysis was performed.

Measurement data were described by mean ± standard deviation (SD; *x* ± *s*). Median was used for data with skewed distribution, and the homogeneity test of variance was used for normal distribution. A *t*-test was used to analyze the variance between two independent sample groups, and a variance analysis was performed for significant tests among multigroups. Logistic regression was used to analyze the risk factors of ROSC and survival to discharge. Regression parameters included age (year), duration of CPR, presence of endotracheal tube before CPA, initial heart rhythm (VT/VF), number of CPR attempts, the dose of epinephrine (contained three categories: 1 ≤ 3 times, 3–5 times, >5 times), and sodium bicarbonate. *p* < 0.05 was considered statistically significant. In this study, the Box-Tidwell method was used to test whether they were linear among the conversion values of continuous independent variables and dependent variables logit. All continuous independent variables should have a linear relationship with the logit transformation value of the dependent variable. If no linear relationship existed between one or more continuous independent variables and the logit transformation value of the dependent variable, the variable was converted into an ordered classification variable. A total of 11 items (i.e., 7 independent variables, 3 interaction items, and 1 intercept item) were included in the linear test model, and the significance level corrected by Bonferroni was 0.0045. *p* < 0.05 was considered statistically significant.

## Results

**Figure 1:**
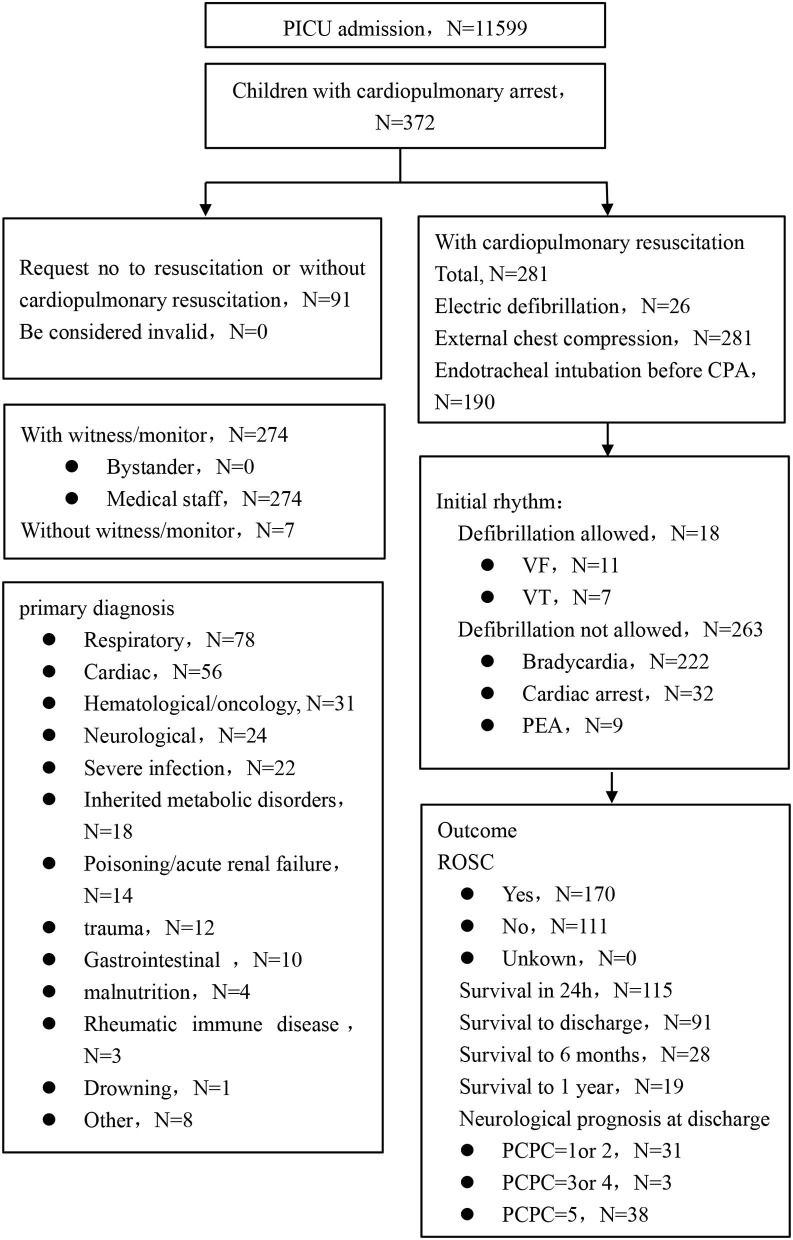
The procedure of inclusion and basic information.

A total of 11,599 cases from 11 PICUs were investigated, and the overall incidence of CPA was 3.2% (372/11,599) with variance ranging from 1.1 to 6.18% among the different centers. Among the 372 children, 281 (75.5%, 281/372) had received CPR (that includes 1 ECPR), and the percentage in each center ranged from 33.3 to 96.5%. In addition, 24.5% of the subjects were requested for DNR (91/372), the ratio in the different centers ranged from 0 to 66.7%. A total of 170 of the 281 children (60.1%) had ROSC, in which the survival in 24 h, survival to hospital discharge, and survival to 6 months and 1 year after discharge were 47.3, 37.4, 11.5, and 7.8%, respectively (Figure 1 and Tables 1, 2). According to previous epidemiological reports from China, the incidence of CPA in the PICU decreased from 5.5 to 2.2% with an increase in CPR ratio from 17.1 to 84.4% (Table 3).

**Table 1:** Distribution and basic information of pediatric intensive care units (PICUs) in current data.

**Hospital participated**	**CPA/PICU admission *n* (%)**	**CPR/PICU admission (%)**	**DNR/PICU admission (%)**	**CPR/CPA (%)**	**DNR/CPA (%)**
Beijing Children's Hospital	60/1,023 (5.87)	38/1,023 (3.68)	22/1,023 (2.1)	38/60 (63.4)	22/60 (36.6)
Guiyang Maternal and child Health Care Hospital	48/777 (6.18)	41/777 (5.28)	7/777 (0.9)	41/48 (85.4)	7/48 (14.6)
Xian Children's Hospital	45/949 (4.74)	45/949 (4.74)	0	45/45 (100)	0
Hunan Children's Hospital	41/1,529 (2.68)	30/1,529 (1.96)	11/1,529 (0.71)	30/41 (73.1)	11/41 (26.9)
Anhui Children's Hospital	40/1,012 (3.95)	33/1,012 (3.26)	7/1,012 (0.69)	33/40 (82.5)	7/40(17.5)
Zhengzhou Children's Hospital	36/1,295(2.78)	25/1,295(1.93)	11/1,295(0.84)	25/36 (69.4)	11/36 (30.6)
Hebei Children's Hospital	29/1,265 (2.29)	28/1,265 (2.21)	1/1,265 (0.07)	28/29 (96.5)	1/29 (3.5)
Jinan Children's Hospital	24/1,134 (2.12)	8/1,134 (0.71)	16/1,134 (1.4)	8/24 (33.3)	16/24 (66.7)
Nanjing Children's Hospital	22/1,031 (2.13)	13/1,031 (1.26)	9/1,031 (0.87)	13/22 (59.1)	9/22 (40.9)
Shandong General Hospital	19/857 (2.22)	16/857 (1.87)	3/857 (0.35)	16/19 (84.2)	3/19(15.8)
Liaocheng General Hospital	8/727 (1.1)	4/727 (0.55)	4/727 (0.55)	4/8 (50)	4/8 (50)
Total number	372/11,599 (3.2)	281/11,599 (2.42)	91/11,599 (0.78)	281/372 (75.5)	91/372 (24.5)

**Table 2:** Major parameters of epidemiology.

CPA rate in PICU	372/11,599 (3.2%)
CPR rate in CPA children	281/372 (75.5%)
ROSC rate	170/281 (60.3%)
Survival in 24 h	115/243 (47.3%)
Survival to hospital discharge	91/243 (37.4%)
Survival after 6 months	28/243 (11.5%)
Survival after 1 year	19/243 (7.8%)

**Table 3:** Epidemiology information of PICUs in previous reports.

**Nation and study period**	**CPA/PICU admission (%)**	**CPR/PICU admission (%)**	**No Resuscitation/**	**CPR/CPA**	**DNR/CPA**
			**PICU admission (%)**	**(%)**	**(%)**
Canada (6), 1997–2002	5.5% (531/9,715)	0.94% (91/9,715)	4.6% (450/9,715)	17.1% (91/531)	82.9% (440/531)
USA, 2009–2013 (4)	2.2% (7,390/329,982)	–	–	–	–
USA, 2011–2013 (3)	–	1.4% (139/10,078)	–	–	–
USA, 2017–2018 (7)	2.2% (45/2,053)	1.9% (38/2,053)	0.3% (7/2,053)	84.4% (38/45)	15.6% (7/45)
UK, 2013–2017 (1)	–	2.2% (1,528/68,114)	–	–	–

Nearly 90% of the children who had CPR were under 8-year old, among whom, 49.5% were <1-year old. In the primary diagnosis, respiratory disease accounted for 28.1%, circulatory for 19.6%, hematologic/neoplastic for 11%, sepsis for 7.8%, poisoning/renal disease for 4.9%, and trauma for 4.3%. Bradycardia was the most common dysrhythmia (79%) when CPA occurred, followed by electrical asystole (11.4%), ventricular tachycardia (VT, 2.5%), and ventricular fibrillation (VF, 3.9%). Then, 67.6% of the children had endotracheal intubation before CPA, and 44.8% had CPR duration of <10 min. In addition, 44.1% of the children were given epinephrine 1–3 times, 43.8% had more than 3 times, and 12.1% were not administered with epinephrine. Moreover, 41.3% of the children were administered sodium bicarbonate during resuscitation (Table 4).

**Table 4:** Basic information of CPA children (*N* = 281).

**Item**	**Number/total (%)**
Age	
29 days−1 year	139 (49.5)
>1–8 years	115 (40.9)
>8–18 years	27 (9.6)
**Gender**
Male	144 (51.2)
**Primary diagnosis**
Respiratory	79 (28.1)
Circulatory	55 (19.6)
Neurological	24 (8.5)
Liver/gastrointestinal	10 (3.6)
Inherited metabolic	18 (6.4)
Hematologic/oncologic	31 (11.0)
Systemic infection/shock	22 (7.8)
poisoning and renal failure	14 (4.9)
trauma	12 (4.3)
Other	16 (5.7)
**Initial heart rhythm**
Bradycardia	222 (79)
Electrical asystole	32 (11.4)
Ventricular tachycardia, VT	7 (2.5)
Ventricular fibrillation, VF	11 (3.9)
Pulseless electrical activity	9 (3.2)
**Endotracheal intubation before CPA**
Yes	190 (67.6)
**Duration of CPR**
0–15 min	159 (56.6)
15–30 min	38 (13.5)
>30 min	84 (29.9)
**Epinephrine**
Yes	247 (87.8)
No	34 (12.1)
**Dose of epinephrine**
1 ≤ ~3 times	124 (44.1)
>3~5 times	34 (12.1)
>5 times	89 (31.7)
**Sodium bicarbonate**
Yes	116 (41.3)
No	165 (58.7)
Time of CPR start
08:00–15:59 am	113 (40.2)
16:00–23:59 pm	98 (34.9)
00:00–07:59 am	70 (24.9)

Table 5 shows the ROSC ratios in the children with respiratory and circulatory diseases as 70.5 and 63.6%, respectively, and in trauma as 50%, whereas the corresponding ROSC ratios in hematologic/neoplastic diseases and poisoning/renal failure were 35.5 and 37.5%. Compared with those who had been endotracheal intubated, children without endotracheal intubation had higher ROSC (70.2% vs. 54.7%, *p* = 0.018), and 87.4% of children with CPR ≤15 min had ROSC while the percentage was only 15.5% in children with CPR >30 min (*p* = 0.000). Differences in epinephrine administration corresponded to different ROSC incidences, such as 1 ≤ 3 times, 3–5 times, and >5 times corresponding to 89.5, 50, and 15.3%, respectively (*p* = 0.000). Children obtained a higher ROSC rate when they received no sodium bicarbonate when compared with those who received such compound (68.5% vs. 49.1%, *p* = 0.001) during resuscitation (Table 5).

**Table 5:** Clinical data of children who achieved the return of spontaneous circulation (ROSC) and survival to discharge.

		ROSC^*^N = 170 number/total (%)	***p***	**Survival to discharge^#^ *N* = 91 number/total (%)**	***p***
Age	29 days−1 year	79/139 (56.8)	0.076	43/126 (34.1)	0.039
	>1–8 years	77/115 (66.9)		42/92 (45.7)	
	>8–18 years	14/27 (51.8)		6/25 (24)	
Gender	Male	87/144 (60.4)	0.977	52/126 (41.3)	0.202
	Female	83/137 (60.6)		39/117 (33.3)	
Primary diagnosis	Respiratory	55/78 (70.5)	0.012	37/67 (55.2)	0.035
	Circulatory	35/56 (62.5)		19/49 (38.8)	
	Neurological	15/24 (62.5)		6/18 (33.3)	
	Liver/gastrointestinal	6/10 (60)		2/8 (25)	
	Inherited metabolic	11/18 (61.1)		5/14 (35.7)	
	Hematologic/oncologic	11/31 (35.5)		6/30 (20)	
	Systemic infection/shock	12/22 (54.5)		5/21 (23.8)	
	Poisoning/renal failure	5/14 (37.5)		5/13 (38.5)	
	trauma	6/12 (50)		2/12 (16.7)	
	Other	14/16 (87.5)		4/11 (36.4)	
Initial heart rhythm	Bradycardia	141/222 (63.5)	0.223	83/194 (42.8)	0.000
	Electrical asystole	17/32 (53.1)		5/24 (20.8)	
	Ventricular tachycardia	2/7 (28.6)		0/7 (0.0)	
	Ventricular fibrillation	6/11 (54.5)		1/10 (10)	
	Pulseless electrical activity	4/9 (44.4)		2/8 (25)	
Endotracheal intubation before CPA	Yes	104/190 (54.7)	0.018	57/168 (33.9)	0.09
	No	66/94 (70.2)		34/75 (45.3)	
Duration of CPR	0–15 min	139/159 (87.4%)	0.000	77/125 (61.6)	0.000
	>15–30 min	18/38 (47.4)		10/36 (27.8)	
	>30 min	13/84 (15.5%)		4/82 (4.9)	
Epinephrine	Yes	140/247 (56.7)		75/217 (34.6)	
	No	30/34 (88.2)		16/26 (61.5)	
	1 ≤ ~3 times	111/124(89.5)	0.000	60/101 (59.4)	0.000
	3~5 times	17/34 (50.0)		10/30 (33.3)	
	>5 times	12/89 (13.5)		5/86 (5.8)	
Sodium bicarbonate		*N* = 170, total = 281		*N* = 91, total = 243	
	Yes	57/116 (49.1)	0.001	28/102 (27.5)	0.006
	No	113/165 (68.5)		63/141 (44.7)	
Number of CPR attempts	1 time	–	–	77/186 (41.4)	0.022
	>1 time	–	–	14/57 (24.6)	
time of CPR start	08:00–15:59 am	72/113 (63.7)	0.663	–	–
	16:00–23:59 pm	57/98 (58.2)		–	
	00:00–07:59 am	41/70 (58.6)		–	

* *Total of 281 cases had ROSC documents*.

# *Total of 243 cases had survival to discharge document*.

*^−^Records missing*.

Table 5 also shows the factors associated with survival after hospital discharge. Children aged 1–8 years had the highest survival to hospital discharge (45.7%), those under 1 year had 34.1%, and those older than 8 years had 20% (*p* = 0.039). The rates of survival to discharge in children with respiratory and circulatory diseases were 45.7 and 33.8%, respectively. Children with bradycardia, electrical asystole, VT, and VF had survival to discharge rates of 42.8, 20.8, 0, and 10%, respectively, which were lower than their corresponding ROSC rates of 63.5, 53.1, 28.6, and 54.5%. This result implied that the survival of children with CPA in the PICU was not only correlated with CPR but also with the severity of the original diseases. The ROSC of children with trauma was 50%, but only 16.7% of them were alive to discharge. Furthermore, children who had only one CPR, with CPR for 0–15 min, epinephrine used for 1–3 times, and received no sodium bicarbonate were prone to obtain a higher survival rate (*p* < 0.05). Although not being intubated prior to CPR was beneficial for children to ROSC, it did not correlate with survival to hospital discharge (*p* = 0.09).

The prognosis of the neurological status at the discharge of the 72 surviving children was assessed by PCPC, which was divided into 3 grades: 1–3, 4, and 5 scores. The results showed that the neurological prognosis was good (1–3 scores) in 47.2% but poor (5 scores) in 51.4%.

A multivariate logistic regression analysis of ROSC revealed that the duration of CPR and the dose of epinephrine were correlated with ROSC, and the duration of CPR, primary diagnosis, number of CPR attempts, and the dose of epinephrine were correlated with survival to hospital discharge. Although the age and presence of endotracheal tube before CPA were evidently relevant to ROSC and survival, they were not dependent on influencing factors (Tables 6, 7).

**Table 6:** Influence factors on the ROSC by multivariate logistic regression analysis.

	***p***	**OR**	**95%CI**
Age (year)	0.947	1	0.991–1.008
Duration of CPR^a^	0.003	0.429	0.244–0.754
Presence of endotracheal tube before CPA	0.26	0.621	0.271–1.423
Dose of epinephrine^b^	0.000	0.241	0.138–0.422
Sodium bicarbonate	0.823	1.089	0.517–2.292

a *indicate a continuous variable*;

b *indicate a classified variable containing 3 levels*.

**Table 7:** Influence factors on survival to discharge by a multivariate logistic regression analysis.

	***p***	**OR**	**95%CI**
Age (year)	0.867	1.001	0.993–1.008
Duration of CPR^a^	0.001	0.305	0.152–0.615
Presence of endotracheal tube before CPA	0.91	0.961	0.479–0.927
VT/VF	0.026	0.084	0.01–0.742
Number of CPR attempts^a^	0.000	0.155	0.063–0.378
Epinephrine	0.006	0.398	0.207–0.765
sodium bicarbonate	0.864	1.425	0.658–3.088

a *indicate a continuous variable*.

## Discussion

### Main Findings

From the multicenter study, we found that the incidence of CPA in PICU in China is 3.2%, the implementation rate of CPR is 75.5%, the ROSC rate is 60.3%, the discharge survival rate is 37.4%, and the percentage of discharged survivors with a PCPC score of 1–3 is 47.2%.

### Interpretation and Comparison With Previous Literature

This study is the only multicenter survey of CPA and CPR in PICU based on the Utstein style across China in the past 10 years. Reports in the recent 5 years have declared that the incidence of CPA in critically ill children in the hospital has decreased from 2 to 6% (8, 9) to 2.2–5.5% (1, 3, 4, 7, 10). In this study, the incidence of CPA in PICU was 3.2%, similar to that observed in the previous studies (7, 11) but was lower than the 5.5% in Zeng's report (9), another multicenter survey in Beijing in 2013. However, a possible decrease in the incidence of CPA in the PICU was difficult to determine because of the evident difference in the study population between the two studies. A variance in incidence existed among different centers ranging from 1.1 to 6.8% probably because of the different primary diagnosis spectrum, severity, and ability of early recognition. In addition, compared with Zeng's report, the current rate of ROSC was slightly lower, while the rate of survival in 24 h post-CPR and survival to discharge were apparently higher (47.3 and 37.4% vs. 35.06 and 28.2%, respectively), implying that the subsequent therapy contributed to the improvement of prognosis. In recently published international reports, the ROSC ratio varied widely from 64.5 to 78% (2, 3, 11), and the survival to discharge rate ranged from 45 to 63% (1, 4, 7), which were better than the results in this study. Thus, more studies on repeated high-quality CPR training, primary disease therapy, and effective advanced life support should be performed to narrow the gap.

In this study, 75.5% of the CPA patients had received CPR. The residual 24.5% of the children were requested to receive DNR, with an obvious variance from 0 to 66.7% among the different centers. DNR was common in critically ill patients, which was dependent on several factors, such as disease, social, and economic reasons. Hon (6) found that 44% of deaths in the ICU in Hong Kong were called for DNR, when CPA had occurred. Although the termination of resuscitation has been discussed frequently, limited literature (12) on pediatric critical illness, except for trauma out-of-hospital, is available. Thus, when and why DNR should be conducted was uncertain. Hence, since DNR is wise and reasonable in some cases, the medical staff should still make the guardians fully understand the benefits and value of CPR to increase the survival rate.

Respiratory, circulatory, hematologic, or oncologic status and infection are common causes for critical diseases (13) and CPA (1, 3, 11) in PICU, because they could induce severe hypoxemia, hypovolemia, acid-base disturbance, and multiple organ disease syndrome. Similar to previous studies (1, 3, 9, 14), our results showed that respiratory and circulatory diseases were the major contributors to CPA. Trauma was rarely mentioned in previous reports on the characteristics of CPA. This study indicated that 4.3% of CPA was associated with trauma. Head injury is the most common site of trauma in children (15). The condition is always aggravated by secondary life-threatening complications, such as increased intracranial pressure, electrolyte imbalance, metabolism disturbance in the myocardium, and inhibition of the respiratory center. Our results showed that the ROSC of trauma was 50%, but only 16.7% had survived to discharge. We made no further analysis about the exact cause of death in trauma, but trauma-associated respiratory (16) and renal injury (14) were attributed to increased mortality.

Epinephrine is the most common medication used during resuscitation. Our results revealed that the survival rate to discharge was poor in children with a dose of epinephrine of more than 5. This finding was similar to Meert's report, in which an inverse correlation between the dose of epinephrine and 12-month survival was reported (17). Prior studies have proved that mortality increased as the CPA and CPR persisted (3, 9, 18). In our opinion, the poor outcomes were actually due to severe illnesses, which caused prolonged CPA and CPR, and clinicians had to administer epinephrine repeatedly.

Sodium bicarbonate is a common treatment for acidosis induced by CPA. However, recent CPR studies in adults and children did not prove the benefit of sodium bicarbonate in improving prognosis, and AHA Guidelines recommend its use only for hyperkalemia, tricyclic antidepressants, or barbiturate poisoning (10, 19). Similar to epinephrine, this study showed that sodium bicarbonate during CPR was negatively related to the survival rate to discharge, and the use of sodium bicarbonate was also due to the severity of the disease and prolonged CPR.

The neurologic outcome after CPR was good (20), and 40–64.3% of the children in the PICU had obtained PCPC scores of 1–3 (2–4). The longer the duration of CPR, the poorer the neurological prognosis. Our results showed that 47.2% of the children scored 1–3, which was different from previous reports abroad. Better cerebral perfusion has been correlated with neurologic outcomes. Therefore, we should increase the quality of CPR by improving training to restore heart function and maintain circulatory stability.

With the pediatric life support program by the AHA launched in China in 2001 and the improvements in critical care, the quality of CPR and prognosis had gradually improved.

## Limitations

This study provides updated and reliable evidence-based data about the epidemiology characteristics of CPA and CPR in the PICU in mainland China. The data are important for Chinese pediatricians and for the world despite the following limitations. First, we did not analyze the PCPC score at 6 months and 1 year after discharge due to limited follow-up information. Given that we lacked baseline PCPC scores (before CPA occurred), we were unable to determine if CPR contributed to a change in the cerebral function. Second, a recall bias may exist despite members promptly recordingall data. Third, post-resuscitation treatment measures were not controlled, which may have affected outcomes.

## Conclusion

The prospective multicenter study described the present situation of CPA and CPR in the PICU of China. The incidences of CPA and CPR were relatively high, but there were significant differences among different centers, which were mainly due to the severity of the disease and the rescue willingness of children's parents. In general, the short-term prognosis of CPR is not satisfactory and needs to be further improved. Duration of CPR and the dose of epinephrine were associated with the difficulty of resuscitation and thus with eventual resuscitation outcome.

## Data Availability Statement

The raw data supporting the conclusions of this article will be made available by the authors, without undue reservation.

## Ethics Statement

This study had been approved by the Ethics Committee of each hospital. Written informed consent to participate in this study was provided by the participants' legal guardian/next of kin.

## Author Contributions

All authors listed have made a substantial, direct, and intellectual contribution to the work and approved it for publication.

## Conflict of Interest

The authors declare that the research was conducted in the absence of any commercial or financial relationships that could be construed as a potential conflict of interest.

## Publisher's Note

All claims expressed in this article are solely those of the authors and do not necessarily represent those of their affiliated organizations, or those of the publisher, the editors and the reviewers. Any product that may be evaluated in this article, or claim that may be made by its manufacturer, is not guaranteed or endorsed by the publisher.
